# Body roundness index as a predictor of 3-month readmission in elderly patients with first-episode acute heart failure

**DOI:** 10.3389/fmed.2026.1897382

**Published:** 2026-08-03

**Authors:** Junfang Ma, Meixiang Han, Fenjuan Chen, Jinying Wang

**Affiliations:** 1 Intensive Care Unit, Shaoxing Second Hospital, Shaoxing, Zhejiang, China; 2 Department of Internal Medicine, Shaoxing Second Hospital, Shaoxing, Zhejiang, China

**Keywords:** 3-month, acute heart failure, body roundness index, elderly patients, readmission

## Abstract

**Aims:**

To determine whether admission body roundness index (BRI), a validated anthropometric metric of central adiposity, independently predicts 3-month all-cause readmission and rehospitalization for acute heart failure (AHF) in elderly patients hospitalized for a first AHF episode.

**Methods:**

In this single-center retrospective cohort study, 317 patients aged >60 years with a first AHF hospitalization were enrolled and stratified by BRI tertiles. The endpoints were all-cause readmission and AHF rehospitalization within 3 months. Patients who died during follow-up were retained in the cohort and censored at the time of death, and associations were analyzed using the cause-specific Cox proportional hazards model. Associations were assessed using multivariable Cox regression, restricted cubic spline analysis, and pre-specified subgroup analyses.

**Results:**

During follow-up, 3 patients (0.95%) died (1 in each tertile at 9, 22, and 45 days) and were censored at the time of death, and 128 patients (40.76%) experienced all-cause readmission and 68 (21.73%) were rehospitalized for AHF. After full adjustment, each 1-unit increment in BRI was associated with a 48% higher risk of all-cause readmission (HR 1.48, 95% CI 1.20–1.83) and a 2.15-fold risk of AHF rehospitalization (HR 2.15, 95% CI 1.55–2.98); risks in the highest tertile significantly exceeded those in the lowest tertile. Restricted cubic spline analysis unmasked striking nonlinear threshold effects: all-cause readmission risk escalated sharply when BRI surpassed 5.40 (HR 2.75, 95% CI 1.98–3.82), whereas AHF rehospitalization risk surged beyond a BRI of 7.05 (HR 2.93, 95% CI 1.34–6.40). Notably, the middle BRI tertile exhibited a markedly lower all-cause readmission risk relative to the lowest tertile (HR 0.38, 95% CI 0.17–0.87), whereas AHF rehospitalization risk did not differ. This divergent pattern mechanistically reconciles the “obesity paradox” by distinguishing a sarcopenic phenotype in the lowest-BRI group from a high-adiposity phenotype in the upper extreme. The direction of association remained consistent across all subgroups, with significant effect modification by smoking status, marital status, NYHA class, and diabetes.

**Conclusion:**

Admission BRI is an independent predictor of 3-month all-cause readmission and AHF rehospitalization in elderly patients with AHF, and may serve as a simple, low-cost tool for early post-discharge risk stratification in this population.

## 1 Introduction

Acute heart failure (AHF) ranks among the most common cardiovascular emergencies in the elderly, with all-cause readmission rates reaching 30%–50% within 3 months of discharge and rehospitalization for recurrent heart failure accounting for roughly 25%–30% of cases (1–3). This dual burden imposes substantial healthcare strain and erodes quality of life. Precisely identifying high-risk elderly individuals during this early “vulnerable window” and deploying targeted interventions is therefore a pivotal step toward improving outcomes.

Central obesity, especially visceral adipose tissue (VAT) accumulation, is an independent risk factor for the development and progression of heart failure (4). Yet, routinely used clinical metrics fail to capture this risk adequately: body mass index (BMI) cannot discriminate fat from muscle (5), while waist circumference (WC) is not adjusted for height (6), leading to misclassification in individuals at the extremes of body size. The body roundness index (BRI), introduced by Thomas et al. (7), incorporates waist circumference and height into an elliptical geometric model and thereby estimates VAT area and body fat percentage more accurately. BRI has already outperformed BMI and WC in predicting metabolic syndrome, diabetes, and incident heart failure (8, 9). Wang et al., drawing on the Kailuan cohort, reported that each standard-deviation increase in BRI was associated with an 18% higher risk of new-onset heart failure (10); Zhang et al. observed a U-shaped relationship between BRI and all-cause mortality in the general population, with an inflection point near 5.5 (11). Critically, all existing evidence concentrates on long-term prognosis or disease-free populations. Whether BRI can distinguish elderly patients with established AHF who carry the highest short-term readmission risk remains unexplored.

The present study aimed to investigate the association of admission BRI with 3-month all-cause readmission and AHF rehospitalization in elderly patients after a first AHF episode, seeking to offer a simple, low-cost risk-stratification tool that can inform individualized discharge planning and follow-up decisions, ultimately improving near-term prognosis.

## 2 Materials and methods

### 2.1 Study population and study design

This was a single-center retrospective cohort study that screened 587 elderly patients with a first episode of AHF at Shaoxing Second Hospital between January 2024 and December 2025. Patients were included if they had a first diagnosis of AHF according to the 2022 AHA/ACC/HFSA guidelines (12), were older than 60 years, and had complete baseline (admission) and follow-up data. We excluded patients with less than 3 months of follow-up, those with concomitant active malignancy, severe infection, or end-stage disease, those who had previously undergone heart transplantation or ventricular assist device implantation, and those who were lost to follow-up during the study. After applying these criteria, 317 patients were ultimately enrolled (Supplementary Figure S1). Three patients (0.95%) died during the 3-month follow-up period (1 in each tertile, T1 at day 22, T2 at day 45, and T3 at day 9). These patients were retained in the analytical cohort and censored at the time of death, consistent with the cause-specific Cox model approach. The study was conducted in accordance with the Declaration of Helsinki, approved by the Ethics Committee of Shaoxing Second Hospital (approval number SXEY-2026-027-001), and informed consent was obtained from all patients.

### 2.2 Exposure variable: BRI

Height (m), body weight (kg), and waist circumference (m) were measured at admission. BRI was calculated as (7): $\mathrm{BRI}=364.2-365.5\times \sqrt{1-{\left(\frac{\mathrm{WC}}{\pi \times \text{Height}}\right)}^{2}}$ .

Patients were categorized into three groups according to BRI tertiles: T1 (low BRI), T2 (middle BRI), and T3 (high BRI).

### 2.3 Terminal event

The primary outcomes were all-cause readmission and AHF rehospitalization within 3 months. All-cause readmission was defined as any rehospitalization within 3 months for any reason. AHF rehospitalization was defined as rehospitalization due to worsening heart failure symptoms. All outcomes were confirmed through the electronic medical record system and telephone follow-up, and only the first event was counted in the analysis.

### 2.4 Covariates

All data were extracted from the electronic medical record system. Demographic characteristics included age, sex, height, waist circumference, marital status, and educational level. Lifestyle variables included smoking history. Clinical data comprised New York Heart Association (NYHA) functional class and comorbidities (coronary heart disease, hypertension, diabetes mellitus, and chronic kidney disease). Laboratory parameters included glycated hemoglobin (HbA1c), total cholesterol (TC), serum creatinine (SCr), albumin (ALB), high-sensitivity C-reactive protein (hs-CRP), and estimated glomerular filtration rate (eGFR). Waist circumference was measured at admission at the end of quiet expiration, at the midpoint between the lower costal margin and the iliac crest along the mid-axillary line, and the average of two measurements was recorded. Age was stratified as ≤75 years and >75 years. Marital status was categorized as married/living with partner, widowed/divorced/separated, and never married, encompassing the full range of social support that may influence health outcomes in this elderly population. Educational level was classified as middle school or below, high school, and college or above.

### 2.5 Statistical analysis

Data management and statistical analyses were performed using the Storm Statistical Platform (Zstats software; www.zstats.net) and R version 4.4.0. Categorical variables are presented as frequencies or percentages and were compared using the chi-squared test or Fisher’s exact test, as appropriate. Normally distributed continuous variables are expressed as mean ± standard deviation and were compared using the independent-samples *t*-test or one-way analysis of variance (ANOVA); non-normally distributed continuous variables are expressed as median (interquartile range) and were compared using the Wilcoxon rank-sum test or the Kruskal–Wallis H test. Survival curves for all-cause readmission and AHF rehospitalization stratified by BRI tertile were generated using the Aalen–Johansen estimator, and differences between groups were assessed with the Gray’s test. Multivariable Cox proportional hazards models were employed to evaluate the association between BRI and adverse outcomes (3-month all-cause readmission and AHF rehospitalization). In the cause-specific framework, patients who died during follow-up were retained in the cohort and censored at the time of death, rather than excluded entirely. This approach avoids the selection bias that would arise from removing decedents while maintaining the standard Cox model structure. Three hierarchically adjusted models were constructed: Model 1 was unadjusted; Model 2 was adjusted for demographic characteristics, lifestyle factors, and clinical data, including age, sex, smoking history, education level, marital status, NYHA class, coronary artery disease, hypertension, diabetes mellitus, and chronic kidney disease; Model 3 was additionally adjusted for laboratory parameters, incorporating total cholesterol, albumin, and eGFR on top of all covariates in Model 2. Notably, serum creatinine was excluded to avoid collinearity with eGFR (variance inflation factor [VIF] > 30 when both were included; all VIFs in the model were < 2.5). HbA1c and hs-CRP were conceptualized as mediators on the causal pathway from adiposity to readmission rather than as confounders; adjusting for mediators would induce overadjustment bias, so they were omitted from the multivariable model. Variance inflation factors were calculated to assess multicollinearity; all VIFs in the Model 3 were below 2.5, indicating no serious collinearity. BRI was entered into the models both as a continuous variable (per 1-unit increase) and as categorical tertiles (with T1 serving as the reference group). Restricted cubic spline (RCS) analysis was performed to explore potential nonlinear dose–response relationships between BRI and the outcomes. Subgroup analyses with formal interaction tests were conducted to examine the consistency of the observed associations. The proportional-hazards assumption was assessed by testing the correlation between Schoenfeld residuals and survival time for each covariate in the cause-specific Cox models. All tests were two-sided, and *p* < 0.05 was considered statistically significant.

## 3 Results

### 3.1 Baseline characteristics

A total of 317 elderly patients with AHF were enrolled and stratified by BRI tertiles: T1 (≤4.18, *n* = 106), T2 (4.18–6.28, *n* = 105), and T3 (>6.28, 106). As shown in Table 1, statistically significant differences were observed across tertiles in age, sex, smoking history, education level, marital status, NYHA class, comorbidities, and laboratory parameters (all *p* < 0.05). Compared with the T1 group, the T3 group had higher proportions of patients aged >75 years, males, ever-smokers, those with low educational attainment, those who were unmarried/widowed/divorced, and those in NYHA class III/IV, as well as higher prevalences of coronary artery disease, hypertension, diabetes mellitus, and chronic kidney disease. In addition, the T3 group exhibited higher levels of HbA1c, total cholesterol, serum creatinine, and hs-CRP, together with lower albumin and eGFR levels.

**Table 1:** Baseline characteristics.

Variable	Total (*N* = 317)	T1 (*n* = 106)	T2 (*n* = 105)	T3 (*n* = 106)	Statistic	*p*
Age, *n* (%)					*χ*^2^ = 90.85	<0.001
≤75 years	187 (59.0)	99 (93.4)	56 (53.3)	32 (30.2)		
>75 years	130 (41.0)	7 (6.6)	49 (46.7)	74 (69.8)		
Gender, *n* (%)					*χ*^2^ = 25.62	<0.001
Female	127 (40.1)	63 (59.4)	37 (35.2)	27 (25.5)		
Male	190 (59.9)	43 (40.6)	68 (64.8)	79 (74.5)		
Smoking history, *n* (%)					*χ*^2^ = 19.13	<0.001
No	175 (55.2)	76 (71.7)	54 (51.4)	45 (42.5)		
Yes	142 (44.8)	30 (28.3)	51 (48.6)	61 (57.5)		
Education level, *n* (%)					*χ*^2^ = 31.63	<0.001
Middle school or below	194 (61.78)	47 (44.76)	66 (63.46)	81 (77.14)		
High school	81 (25.80)	33 (31.43)	26 (25.00)	22 (20.95)		
College or above	39 (12.42)	25 (23.81)	12 (11.54)	2 (1.90)		
Marital status, *n* (%)					*χ*^2^ = 30.91	<0.001
Married/living with partner	160 (50.5)	69 (65.1)	59 (56.2)	32 (30.2)		
Widowed/divorced/separated	89 (28.1)	26 (24.5)	26 (24.8)	37 (34.9)		
Never married	68 (21.5)	11 (10.4)	20 (19.0)	37 (34.9)		
NYHA class, *n* (%)					*χ*^2^ = 50.88	<0.001
II	88 (27.8)	54 (50.9)	22 (21.0)	12 (11.3)		
III	160 (50.5)	38 (35.8)	64 (61.0)	58 (54.7)		
IV	69 (21.8)	14 (13.2)	19 (18.1)	36 (34.0)		
Coronary artery disease, *n* (%)					*χ*^2^ = 41.08	<0.001
No	156 (49.2)	77 (72.6)	48 (45.7)	31 (29.2)		
Yes	161 (50.8)	29 (27.4)	57 (54.3)	75 (70.8)		
Hypertension, *n* (%)					*χ*^2^ = 24.51	<0.001
No	87 (27.4)	47 (44.3)	24 (22.9)	16 (15.1)		
Yes	230 (72.6)	59 (55.7)	81 (77.1)	90 (84.9)		
Diabetes mellitus, *n* (%)					*χ*^2^ = 51.68	<0.001
No	164 (51.7)	84 (79.2)	48 (45.7)	32 (30.2)		
Yes	153 (48.3)	22 (20.8)	57 (54.3)	74 (69.8)		
Chronic kidney disease, *n* (%)					*χ*^2^ = 6.10	0.047
No	249 (78.5)	93 (87.7)	86 (81.9)	70 (66.0)		
Yes	68 (21.5)	13 (12.3)	19 (18.1)	36 (34.0)		
HbA1c %, M (Q₁, Q₃)	7.10 (6.20, 8.20)	6.15 (5.50, 6.60)	7.30 (6.80, 7.90)	8.55 (7.20, 9.57)	*χ*^2^ = 78.38	<0.001
Total cholesterol, mmol/L	4.60 (4.00, 5.10)	4.40 (3.80, 4.80)	4.70 (4.00, 5.20)	4.80 (4.30, 5.10)	*χ*^2^ = 14.28	0.001
Serum creatinine, μmol/L	85.12 ± 16.60	76.82 ± 16.84	83.65 ± 12.26	94.88 ± 15.13	*F* = 30.71	<0.001
Albumin, g/L, mean ± SD	37.96 ± 3.13	39.51 ± 3.00	37.97 ± 2.87	36.40 ± 2.72	*F* = 33.87	<0.001
eGFR, M (Q₁, Q₃)	74.70 (62.30, 88.10)	82.25 (68.20, 95.05)	76.10 (66.30, 89.40)	65.80 (56.82, 77.47)	*χ*^2^ = 43.28	<0.001
hs-CRP, mg/L, M (Q₁, Q₃)	3.20 (1.80, 5.20)	1.60 (1.20, 2.20)	3.40 (2.50, 4.60)	5.85 (4.53, 7.70)	*χ*^2^ = 176.75	<0.001
All-cause readmission, *n* (%)					*χ*^2^ = 13.01	0.001
No	189 (59.6)	64 (60.4)	75 (71.4)	50 (47.2)		
Yes	128 (40.4)	42 (39.6)	30 (28.6)	56 (52.8)		
AHF rehospitalization, *n* (%)					*χ*^2^ = 18.73	<0.001
No	248 (78.2)	83 (78.3)	95 (90.5)	70 (66.0)		
Yes	68 (21.5)	22 (20.8)	10 (9.5)	36 (34.0)		
Death during follow-up, *n* (%)					*χ*^2^ = 0.02	0.99
No	314 (99.1)	105 (99.1)	104 (99.0)	105 (99.1)		
Yes	3 (0.9)	1 (0.9)	1 (1.0)	1 (0.9)		

eGFR, estimated glomerular filtration rate; hs-CRP, high-sensitivity C-reactive protein; NYHA, New York Heart Association. “Never married” denotes individuals who have never entered into marriage, which is distinct from “widowed/divorced/separated” (individuals who were previously married but are no longer in a marital union).

### 3.2 Clinical outcomes

Within 3 months of follow-up, 3 patients (0.95%) died during follow-up and were censored at the time of death (1 in each tertile at 9, 22, and 45 days), and 128 patients (40.4%) experienced all-cause readmission and 68 (21.5%) experienced AHF rehospitalization. Cumulative incidence analysis (Supplementary Figure S2) demonstrated clear separation of the cumulative incidence curves across BRI tertiles. For all-cause readmission (Supplementary Figure S2A), the 3-month cumulative readmission incidence rates were 39.6% in T1 (low BRI, ≤4.18), 28.6% in T2 (middle BRI, 4.18–6.28), and 52.8% in T3 (high BRI, >6.28). The T2 group exhibited a relatively favorable short-term outcome, whereas the T3 group maintained the highest cumulative incidence throughout the entire follow-up period. The Gray’s test confirmed a statistically significant difference among the three groups (*p* = 0.001). For AHF rehospitalization (Supplementary Figure S2B), the 3-month cumulative rehospitalization incidence rates were 20.8%, 9.5%, and 34.0% in T1, T2, and T3, respectively. The separation of incidence curves was even more pronounced for this endpoint; T2 again carried the lowest risk of AHF rehospitalization (only 9.6%), while T3 had the highest rehospitalization rate (34.3%). The Gray’s test likewise confirmed a highly significant intergroup difference (*p* < 0.001).

### 3.3 Relationship between BRI and outcomes

We investigated whether BRI served as an independent predictor of adverse outcomes in elderly patients with AHF using multivariable-adjusted cause-specific Cox proportional hazards regression analyses; the results are summarized in Table 2. The proportional-hazards assumption was satisfied for all covariates in the cause-specific Cox models, as indicated by the Schoenfeld residual global test (*χ*^2^ = 5.2, *p* = 0.61) and individual covariate tests (all *p* > 0.05), supporting the validity of the Cox model estimates. For all-cause readmission, when analyzed as a continuous variable, BRI was significantly and positively associated with the risk of all-cause readmission in the unadjusted model (Model 1: HR 1.49, 95% CI 1.33–1.68), the partially adjusted model (Model 2: HR 1.65, 95% CI 1.39–1.95), and the fully adjusted model (Model 3: HR 1.48, 95% CI 1.20–1.83) (all *p* < 0.001). In the categorical analysis by BRI tertiles, compared with T1, the T3 group had a nearly 7-fold higher risk of all-cause readmission in Model 3 (HR 6.50, 95% CI 2.51–17.04, *p* < 0.001). Notably, the T2 group exhibited a protective association (Model 3: HR 0.38, 95% CI 0.17–0.87, *p* = 0.023). For AHF rehospitalization, each 1-unit increase in BRI was associated with a significantly higher risk across all models, with the highest risk observed in the fully adjusted Model 3 (HR 2.15, 95% CI 1.55–2.98, p < 0.001). In the categorical analysis, compared with T1, the T3 group had a significantly elevated risk of AHF rehospitalization in Model 3 (HR 6.57, 95% CI 1.82–23.58, *p* = 0.007), whereas the T2 group did not differ significantly from T1.

**Table 2:** Association between BRI and 3-month adverse outcomes using cause-specific cox proportional hazards models.

Variables	Model1		Model2		Model3	
	HR (95% CI)	*p*	HR (95% CI)	*p*	HR (95% CI)	*p*
All-cause readmission						
BRI	1.49 (1.33 ~ 1.68)	<0.001	1.65 (1.39 ~ 1.95)	<0.001	1.48 (1.20 ~ 1.83)	<0.001
BRI tertiles						
T1	1.00 (Reference)		1.00 (Reference)		1.00 (Reference)	
T2	0.75 (0.47 ~ 0.94)	0.045	0.45 (0.23 ~ 0.90)	0.024	0.42 (0.18 ~ 0.96)	0.038
T3	11.58 (6.23 ~ 21.52)	<0.001	7.96 (3.44 ~ 18.42)	<0.001	6.50 (2.51 ~ 17.04)	<0.001
AHF rehospitalization						
BRI	1.21 (1.07 ~ 1.38)	0.003	1.56 (1.25 ~ 1.94)	<0.001	2.15 (1.55 ~ 2.98)	<0.001
BRI tertiles						
T1	1.00 (Reference)		1.00 (Reference)		1.00 (Reference)	
T2	1.13 (0.61 ~ 2.09)	0.696	0.93 (0.39 ~ 2.21)	0.866	1.60 (0.55 ~ 4.70)	0.38
T3	2.39 (1.29 ~ 4.41)	0.005	2.62 (1.04 ~ 6.62)	0.041	6.57 (1.82 ~ 23.58)	0.004

Model1: crude.

Model2: adjust: age, sex, smoking history, education level, marital status, NYHA class, coronary artery disease, hypertension, diabetes mellitus, chronic kidney disease.

Model3: adjust: age, sex, smoking history, education level, marital status, NYHA class, coronary artery disease, hypertension, diabetes mellitus, chronic kidney disease, total cholesterol, albumin, eGFR.

BRI, body roundness index; AHF, acute heart failure.

### 3.4 RCS model analysis

The RCS analysis revealed nonlinear associations of BRI with both all-cause readmission and AHF rehospitalization (Supplementary Figure S3). Threshold effect analysis (Table 3) identified clear inflection points for each outcome. For all-cause readmission, the inflection point was 5.40: below this threshold, no significant association was observed (HR 0.87, 95% CI 0.67–1.12, *p* = 0.279), whereas at BRI ≥ 5.40 the risk rose significantly (HR 2.75, 95% CI 1.98–3.82, *p* < 0.001). For AHF rehospitalization, the inflection point was 7.05: no significant association was evident below this value (HR 1.00, 95% CI 0.81–1.23, *p* = 0.999), whereas at BRI ≥ 7.05 the risk increased sharply (HR 2.93, 95% CI 1.34–6.40, *p* = 0.007). Likelihood ratio tests confirmed the significance of these threshold effects (all-cause readmission *p* < 0.001, AHF rehospitalization *p* = 0.005).

**Table 3:** Threshold effect analysis of BRI on 3-month adverse outcomes using cause-specific cox models.

Outcome	Effect	*p*
All-cause readmission		
Model 1 fitting model by standard linear regression	1.49 (1.33–1.68)	<0.001
Model 2 fitting model by two-piecewise linear regression		
Inflection point	5.40	
<5.40	0.87 (0.67–1.12)	0.279
≥5.40	2.75 (1.98–3.82)	<0.001
P for likelihood test		<0.001
AHF rehospitalization		
Model 1 fitting model by standard linear regression	1.21 (1.07–1.38)	0.003
Model 2 fitting model by two-piecewise linear regression		
Inflection point	7.05	
<7.05	1.00 (0.81–1.23)	0.999
≥7.05	2.93 (1.34–6.40)	0.007
P for likelihood test		0.005

AHF, acute heart failure.

### 3.5 Subgroup analysis

Subgroup analysis demonstrated that the positive associations of BRI with all-cause readmission and AHF rehospitalization were generally consistent across all examined subgroups (Supplementary Figure S4). Interaction tests identified significant effect modification for all-cause readmission by smoking history (*p* = 0.010), marital status (*p* = 0.006), NYHA class (*p* = 0.008), and diabetes mellitus (*p* = 0.013). For AHF rehospitalization, marital status (*p* = 0.044), NYHA class (*p* = 0.007), and chronic kidney disease (*p* = 0.013) potentially modified the strength of the association. No reversal of effect direction was observed in any subgroup, indicating that the predictive value of BRI exhibits satisfactory robustness across different patient populations.

### 3.6 Sensitivity analysis

To verify the robustness of the primary findings, a sensitivity analysis was conducted using BRI quartiles in place of tertiles. Compared with the lowest quartile group, the highest BRI quartile groups exhibited significantly elevated risks of both all-cause readmission and AHF rehospitalization, with a graded increase in risk across ascending BRI categories. These results were fully consistent with the main analysis, confirming the robustness of the association between BRI and adverse outcomes (Supplementary Table S1).

## 4 Discussion

The relationship between obesity and heart failure extends far beyond simple hemodynamic overload, reflecting the systemic consequences of adipose tissue heterogeneity and microenvironmental remodeling (13). In elderly patients with heart failure, the fundamental limitation of BMI lies in its inability to distinguish fat accumulation from muscle loss, which is a hallmark of age-related sarcopenia (14). Although WC captures central adiposity, its lack of adjustment for height introduces bias among individuals at the extremes of body size (15). The BRI adopted in the present study integrates waist circumference and height into an elliptical geometric model, which may approximate visceral adipose tissue area more closely than waist circumference alone, although this theoretical advantage requires direct validation against imaging-based fat measurements. Previous work from the Kailuan cohort has established BRI as a predictor of incident heart failure (10); however, the current study is the first to extend its prognostic utility to a fundamentally different pathophysiological context: the short-term “vulnerable window” following discharge after a first acute heart failure episode in the elderly. Our central finding is that admission BRI independently predicts 3-month all-cause readmission and AHF rehospitalization, displaying clear nonlinear threshold effects at 5.40 and 7.05. This observation advances BRI from a marker of long-term risk to a clinical tool for early post-discharge decompensation risk stratification.

Elevated BRI increases short-term readmission risk through four interconnected mechanisms. First, visceral fat accumulation raises intra-abdominal pressure, displaces the diaphragm upward, and reduces lung compliance (16), which directly worsens the pulmonary congestion and dyspnea already present in heart failure (17). Central obesity also expands blood volume and activates the renin-angiotensin-aldosterone system, increasing both cardiac preload and afterload (18). For an elderly patient with AHF whose left ventricular reserve is already severely compromised, this dual hemodynamic burden can readily push the circulation from compensation into decompensation (19). Second, hypertrophic visceral adipocytes under hypoxic stress attract macrophages and shift them toward a pro-inflammatory phenotype, leading to a burst of cytokines such as interleukin-6 and tumor necrosis factor-alpha while suppressing adiponectin release (20, 21). The markedly higher hs-CRP levels observed in the high-BRI group provide direct clinical evidence for this adipose-inflammatory axis, and the resulting chronic low-grade systemic inflammation accelerates myocardial fibrosis and adverse remodeling (22, 23). Third, visceral adiposity-driven insulin resistance forces the myocardium to rely more on less efficient glucose utilization rather than efficient fatty acid oxidation, thereby impairing contractile efficiency, while also enhancing renal sodium reabsorption to worsen volume overload (24, 25). The significantly elevated HbA1c in the high-BRI group highlights this metabolic-cardiac pathway. Fourth, epicardial adipose tissue, which is functionally homologous to visceral fat, releases pro-inflammatory and pro-fibrotic factors directly into the coronary circulation and underlying myocardium through paracrine pathways, delivering a regional insult to an already vulnerable heart (26, 27). These four mechanisms do not operate in isolation; they reinforce one another in a positive feedback loop in which inflammation worsens insulin resistance, insulin resistance promotes further fat accumulation, and the expanding fat mass elevates intraventricular filling pressure. Once organ compensatory reserves are exhausted, this vicious cycle culminates clinically in readmission or AHF rehospitalization.

The nonlinear threshold effects observed in this study carry important pathophysiological significance. The risks of all-cause readmission and AHF rehospitalization surged sharply once BRI exceeded 5.40 and 7.05, yielding hazard ratios of 2.75 and 2.93, respectively, whereas below these thresholds no significant association was evident. This pattern closely parallels the critical phase transition from metabolically healthy obesity to metabolically unhealthy obesity. Below the threshold, adipose tissue can still maintain safe lipid storage and secrete adequate anti-inflammatory factors (28); once visceral fat accumulation surpasses the body’s compensatory capacity, adipocytes undergo hypertrophy, hypoxia, and necrosis, transforming adipose tissue from a metabolic buffer into a source of systemic inflammation and excessive RAAS activation (21, 29). It is precisely this nonlinear dynamic shift that allows even a marginal increase in visceral fat beyond the threshold to amplify risk several-fold. Notably, in our study the middle BRI tertile (T2) showed the lowest risk of all-cause readmission. We suspect this does not reflect a genuine protective effect of BRI per se, but rather arises from the distinct pathophysiological profiles of the low-BRI (T1) and high-BRI (T3) groups. Patients in the low-BRI group tended to be leaner and had fewer cardiometabolic comorbidities; nevertheless, their readmission risk was higher than that of the middle-BRI group. This seemingly paradoxical finding may reflect the “obesity paradox” or residual confounding by unmeasured factors such as frailty, nutritional status, or social support, which warrant further investigation (30). In the high-BRI group, although albumin levels were low, the markedly elevated hs-CRP suggests that this hypoalbuminemia was attributable more to dilutional effects driven by chronic inflammation and to suppressed albumin synthesis resulting from heart failure-related hepatic congestion, rather than to malnutrition or cachexia (31, 32). Consequently, the middle-BRI group, which avoided the unfavorable influences of both extreme phenotypes, exhibited a relatively favorable readmission risk.

The clinical value of any prognostic tool ultimately rests on how it compares with existing risk scores and whether it can be smoothly translated into real-world practice. Widely used readmission risk scores such as the LACE index and the HOSPITAL score depend on multiple parameters or complex algorithms, which limits their applicability in time-sensitive bedside settings like the emergency department or at admission (33, 34). BRI, by contrast, requires only three routine anthropometric measurements, namely height, weight, and waist circumference, and can be calculated within seconds without the need for blood sampling, specialized equipment, or prior medical records (35). This low barrier allows it to be seamlessly embedded into the admission nursing assessment as an initial screening filter for risk triage. More importantly, the nonlinear threshold effects uncovered in this study provide clear nodes for stratified intervention. Patients with a BRI exceeding 5.40 should trigger an intensified discharge plan that includes early heart failure clinic follow-up, medication adherence monitoring, and sodium restriction education. Those with a BRI above 7.05 should be regarded as a very-high-risk subgroup; in addition to the measures above. Whether targeted pharmacological therapies such as SGLT2 inhibitors or GLP-1 receptor agonists could further reduce readmission risk in this high-BRI population is an important question that merits exploration in prospective randomized trials, as suggested by the encouraging results of the SUMMIT trial with tirzepatide in obese HFpEF patients (36). Furthermore, the subgroup interactions revealed that marital status (living alone), NYHA class III/IV, and diabetes significantly modified the strength of the association. This suggests that integrating biological indicators with social determinants of health may be more clinically actionable than single-dimension scoring: an elderly patient with AHF who lives alone and has an elevated BRI faces a dually amplified risk of readmission.

In our refined analysis, we retained the three deaths and censored them at the time of death using a cause-specific Cox model, rather than excluding them, to mitigate selection bias. This yielded slightly more conservative readmission hazard estimates (e.g., all-cause readmission T3 vs. T1: HR 6.5 vs. 7.66 in the original analysis), while the core conclusion that higher BRI nonlinearly increases readmission risk remained robust. Several limitations should be acknowledged. The single-center retrospective design is the primary constraint. Despite the cause-specific approach, the small number of deaths (*n* = 3) limits precision, and this model does not estimate cumulative incidence under competing risks as directly as a Fine–Gray model would; multicenter validation with larger event counts is needed. The lack of gold-standard body composition measurements, such as dual-energy X-ray absorptiometry or bioelectrical impedance analysis, precluded separation of visceral and subcutaneous fat and independent quantification of muscle mass (37), limiting mechanistic interpretation. The BRI inflection points observed here require external validation before extrapolation to other populations or clinical decision-making. Finally, head-to-head comparisons with BMI, waist circumference, and a body shape index, as well as net reclassification improvement and integrated discrimination improvement analyses, were not performed. Therefore, we cannot claim that BRI is superior to these simpler indices; rather, BRI should be considered an alternative anthropometric tool whose incremental value requires formal quantification in future studies.

## 5 Conclusion

In conclusion, admission BRI is an independent predictor of 3-month all-cause readmission and AHF rehospitalization in elderly patients with acute heart failure, exhibiting a nonlinear dose–response relationship with inflection points at 5.40 and 7.05, respectively. BRI may serve as a simple, low-cost risk stratification tool in emergency and admission settings, although its clinical thresholds require validation through multicenter external studies.

## Data Availability

The raw data supporting the conclusions of this article will be made available by the authors, without undue reservation.
