# Supplementary material for: Body roundness index as a predictor of 3-month readmission in elderly patients with first-episode acute heart failure
Source: Front Med (Lausanne). 2026 Aug 3;13:1897382. doi: 10.3389/fmed.2026.1897382 (PMC13477963; doi:10.3389/fmed.2026.1897382)
Supplement: Supplementary file 1 [file Data_Sheet_1.docx]

Supplementary Material

**Supplementary Figure 1.** Flowchart of inclusion of participants.

**Supplementary Figure 2.** Cumulative incidence analysis. (A) All-cause readmission, (B) AHF rehospitalization

**Supplementary Figure 3.** RCS analysis. (A) All-cause readmission, (B) AHF rehospitalization

**Supplementary Figure 4.** Subgroup analysis. (A) All-cause readmission, (B) AHF rehospitalization
