# Supplementary material for: Body roundness index as a predictor of 3-month readmission in elderly patients with first-episode acute heart failure
Source: Front Med (Lausanne). 2026 Aug 3;13:1897382. doi: 10.3389/fmed.2026.1897382 (PMC13477963; doi:10.3389/fmed.2026.1897382)
Supplement: Supplementary file 2 [file Table_1.docx]

**Supplementary Table 1.** Association between **BRI quartile** and 3-month adverse outcomes using cause-specific Cox proportional hazards models

| **Variables** | **Model1** | | **Model2** | | **Model3** | |
| --- | --- | --- | --- | --- | --- | --- |
|  | **HR (95%CI)** | **P** | **HR (95%CI)** | **P** | **HR (95%CI)** | **P** |
| **All-cause readmission** |  |  |  |  |  |  |
| **BRI quartile** |  |  |  |  |  |  |
| **T1** | **1.00 (Reference)** |  | **1.00 (Reference)** |  | **1.00 (Reference)** |  |
| **T2** | **0.72 (0.41 ~ 1.26)** | **0.248** | **0.58 (0.29 ~ 1.17)** | **0.128** | **0.57 (0.35 ~ 1.21)** | **0.072** |
| **T3** | **1.02 (0.62 ~ 1.68)** | **0.931** | **0.60 (0.29 ~ 1.24)** | **0.169** | **0.43 (0.21 ~ 1.18)** | **0.067** |
| **T4** | **3.45 (2.16 ~ 5.50)** | **<.001** | **4.12 (1.79 ~ 9.48)** | **<.001** | **2.81 (1.69 ~ 5.03)** | **0.014** |
| **AHF rehospitalization** |  |  |  |  |  |  |
| **BRI quartile** |  |  |  |  |  |  |
| **T1** | **1.00 (Reference)** |  | **1.00 (Reference)** |  | **1.00 (Reference)** |  |
| **T2** | **0.73 (0.33 ~ 1.65)** | **0.453** | **1.15 (0.42 ~ 3.13)** | **0.786** | **1.44 (0.46 ~ 4.51)** | **0.575** |
| **T3** | **0.96 (0.46 ~ 2.01)** | **0.922** | **1.75 (0.62 ~ 4.98)** | **0.294** | **3.81 (0.52 ~ 18.26)** | **0.132** |
| **T4** | **2.34 (1.27 ~ 4.30)** | **0.006** | **10.28 (2.86 ~ 36.94)** | **<.001** | **12.32 (4.77 ~ 20.35)** | **<.001** |

Model1: Crude;

Model2: Adjust: Age, Sex, Smoking History, Education Level, Marital Status, NYHA Class, Coronary Artery Disease, Hypertension, Diabetes Mellitus, Chronic Kidney Disease.

Model3: Adjust: Age, Sex, Smoking History, Education Level, Marital Status, NYHA Class, Coronary Artery Disease, Hypertension, Diabetes Mellitus, Chronic Kidney Disease, Total Cholesterol, Albumin, eGFR.

BRI, body roundness index; AHF, acute heart failure
